# Recurrent Pouchitis Uncovering De Novo Celiac Disease

**DOI:** 10.1155/crgm/2060709

**Published:** 2025-06-13

**Authors:** Chakib Khoury, Rebal Nahas, Emanuel Youssef Dib, Karam Karam, Elias Fiani

**Affiliations:** ^1^Department of Internal Medicine, University of Balamand, Beirut, Lebanon; ^2^Department of Gastroenterology, University of Balamand, Beirut, Lebanon

**Keywords:** celiac disease, chronic antibiotic-resistant pouchitis, ulcerative colitis

## Abstract

We present the case of a 39-year-old woman with a history of severe ulcerative colitis (UC) that was refractory to 5-aminosalicylates, corticosteroids, and biologics, and who subsequently underwent total colectomy with ileal pouch–anal anastomosis (IPAA). She developed chronic antibiotic-refractory pouchitis (CARP) characterized by recurrent abdominal pain, cramping, and diarrhea unresponsive to standard treatments. A comprehensive workup, including testing for anti-tissue transglutaminase IgA antibodies, led to the diagnosis of de novo celiac disease, confirmed by endoscopic and histopathologic findings. Initiation of a gluten-free diet resulted in the resolution of symptoms, with no relapse observed during a 9-month follow-up. Our case highlights the importance of considering secondary etiologies such as celiac disease in patients with chronic refractory pouchitis and emphasizes the need for tailored management strategies.

## 1. Introduction

Pouchitis, an inflammatory condition affecting the ileal pouch created after proctocolectomy, is a common complication in patients who have undergone ileal pouch–anal anastomosis (IPAA) [1]. Despite its prevalence, the pathogenesis of pouchitis remains poorly understood, with the majority of cases being idiopathic [2]. While most cases of acute pouchitis respond well to antibiotics, chronic antibiotic-refractory pouchitis (CARP) presents a significant therapeutic challenge. CARP is often investigated for secondary causes, as up to 30% of cases may be attributed to identifiable factors such as infections, ischemia, or other inflammatory conditions, including Crohn's disease [3]. The occurrence of de novo celiac disease following IPAA is exceedingly rare, with only a few cases documented in the literature [4]. Ulcerative colitis (UC) and celiac disease are both immune-mediated enteropathies. A bidirectional relationship has been suggested between UC and celiac disease, whereby mucosal transcriptomics highlight IncRNAs implicated in UC and celiac disease. This case report discusses a patient with a history of severe UC refractory to multiple lines of treatment, who developed CARP after IPAA. Despite standard management, her symptoms persisted, leading to further investigation and the eventual diagnosis of celiac disease.

This case underscores the need for a comprehensive evaluation of patients with CARP to identify the underlying conditions that may contribute to persistent symptoms and offer insight into the management of such complex cases.

## 2. Case Report

We present the case of a 39-year-old nonsmoking woman known to have severe UC unresponsive to 5-aminosalicylates (ASA), corticosteroids (prednisone), nor biologics (infliximab), status posttotal colectomy with IPAA complicated by CARP manifesting as recurrent abdominal pain with cramping and diarrhea accompanied by mild fevers unresponsive to standard medical management including once daily (OD) suppository mesalamine 1 g and OD oral ASA.

Medical history was remarkable for UC diagnosed 5 years prior to presentation by positive perinuclear antineutrophil cytoplasmic antibodies (p-ANCAs) and negative anti-*Saccharomyces cerevisiae* antibodies (ASCAs) correlated with colonoscopy revealing infiltration of the mucosa and submucosa crypt abscesses as well as crypt branching and shortening. There were no signs of metaplasia nor blunting of villi. She also suffered from gastroesophageal reflux disease, diagnosed using upper-gastrointestinal endoscopy with biopsies retrieved from the lower esophageal sphincter and descending duodenum, which showed no dysplasia, blunting of villi, or intraepithelial lymphocytes. Reflux symptoms are well managed with proton-pump inhibitors.

Surgical history included her uncomplicated total colectomy 3 years ago and adenoidectomy during childhood. Her family history was negative, and she has no known food or drug allergies including lactose intolerance and gluten sensitivity. She does not smoke or use illicit drugs.

A standard initial workup consisting of complete blood count, basic metabolic panel, and liver functional tests showed only mild anemia with leukocytosis, and stool studies came back negative for *C. difficile* and cytomegalovirus antigens.

**Figure 1:**
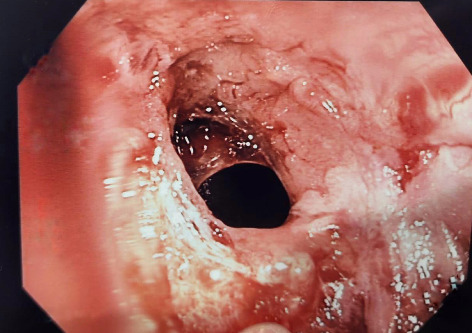
Endoscopic features of active pouchitis, including diffuse erythema, friability, granularity, exudates, erosions, and ulcerations.

**Figure 2:**
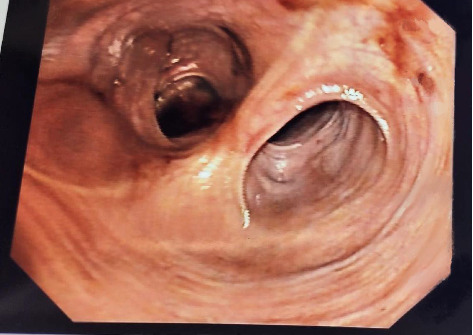
Endoscopic view of the J pouch with an owl's eye appearance.

**Figure 3:**
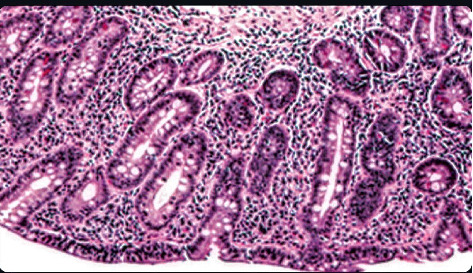
H&E staining of duodenal biopsies revealing scattered intraepithelial lymphocytosis and diffuse villous atrophy.

Further investigation included testing for anti-tissue transglutaminase (TTG) IgA antibodies, which yielded positive. This prompted immediate endoscopy and pouchoscopy with biopsy from the second portion of the duodenum (Figures 1 and 2) that displayed active pouchitis, and histopathology revealed scallop mucosa, crypt hypertrophy, and blunting of villi, confirming active celiac pathology (Figure 3). Of note, the histopathologic evaluation was performed by a gastrointestinal pathology specialist.

The patient was given supportive treatment and started on a gluten-free diet with excellent adherence. She reported resolution of symptoms with no relapse on 6- and 9-month follow-up. Repeat celiac serologies on the 12-month follow-up showed normal levels.

The patient's histopathology records predating her total colectomy by 1 month displayed pancolitis with no signs of celiac, as well as negative celiac serology. This suggests that the celiac disease manifested de novo after undergoing the IPAA.

## 3. Discussion

### 3.1. Background Information

Pouchitis as a disease was first described by Kock et al. in 1977 as a nonspecific inflammatory condition affecting the ileal pouch of patients that underwent proctocolectomy, commonly presenting as increased stool frequency, urgency, incontinence, and pelvic discomfort [5]. Proctocolectomy with IPAA is the ideal approach for patients with familial adenomatous polyposis (FAP) and UC with dysplasia or refractory to medical management [6]. The most common complication of patients with UC post-IPAA surgery is pouchitis, with one study reporting it occurs in up to 59% of patients [7]. Most patients respond positively to antibiotics, but 5%–19% of cases develop chronic or relapsing symptoms [8]. Pouchitis as a disease does not have an official classification yet; however, it can be categorized by disease course and by response to treatment. Acute pouchitis lasts for ≤ 4 weeks, while chronic cases should last > 4 weeks. In terms of recurrence, we can broadly group pouchitis as infrequent (< 3 episodes/year), relapsing (1–3 episodes/year), and continuous. Cases can either be antibiotic-responsive, meaning the resolution of symptoms in < 2 weeks with monotherapy, antibiotic-dependent (i.e., requiring maintenance therapy to achieve remission), and antibiotic-refractory (i.e., not responding to standard therapy) [6]. CARP refers to cases that fail a 4-week monotherapy (metronidazole or ciprofloxacin) and require extensive prolonged therapy consisting of ≥ 2 antibiotics, ASA, corticosteroids, or oral immunomodulators [8].

It is also speculated that genetic susceptibility plays a role in the development of pouchitis. Genetic polymorphism of interleukin-1 receptor antagonists and NOD2/CARD15 has been linked with a higher risk of developing acute or chronic pouchitis, according to immunogenetic studies [9, 10].

Most cases of pouchitis are labeled as idiopathic, as its etiology and pathogenesis are not fully understood. Pouchitis with known etiologies are categorized as “secondary pouchitis” [3]. It is important to thoroughly investigate patients with CARP in search of secondary etiologies. Around 20%–30% of cases of CARP have identifiable secondary causes [8]. They may be attributed to intestinal dysbiosis or fecal stasis inducing bacterial overgrowth; causative agents such as *C. difficile*, *Candida*, or CMV; use of nonsteroidal anti-inflammatory drugs (NSAIDs); ischemia; or inflammatory conditions such as Crohn's or UC recurrence [11, 12]. Due to the high success rate of antibiotic treatment on pouchitis, bacterial pouchitis is the most accepted common etiology.

### 3.2. Typical Presentation and Diagnosis

People with pouchitis have variable clinical presentations. Pouchitis can be suspected in patients with abdominal cramps accompanied by increased stool frequency, urgency, day- or night-time fecal incontinence or seepage, or tenesmus [6]. However, symptoms are insufficient in conclusively diagnosing pouchitis, as its nonspecific presentation resembles other inflammatory conditions such as Crohn's disease, cuffitis, and irritable pouch syndrome (IPS), to name a few; thus, one must employ laboratory and endoscopic investigation including biopsy for histological assessment [8].

The most well-recognized diagnostic tool for the assessment of pouch inflammation is the Pouchitis Disease Activity Index (PDAI), which takes into account symptoms, endoscopy findings, and histology (all scored from 0 to 6 points). Pouchitis can be diagnosed when the score is greater or equal to 7 [1]. However, the PDAI is not specific enough as other inflammatory conditions can elevate the score (Crohn's, UC, IPS, etc.). Nonetheless, a study reported that 25% of the cases with clinical symptoms of pouchitis but a PDAI score below 7 failed to respond to empiric antibiotic therapy, and so they classified them as having IPS [13].

A careful history should be investigated; rectal bleeding is uncommon to pouchitis, and it points to other disorders (i.e., IPS and cuffitis). The use of NSAIDs should also be investigated as it has been theorized to cause inflammatory flares by exacerbating colonic injury while hindering healing [8].

Laboratory studies should include a complete blood panel, a basic metabolic panel, and liver functional tests. Elevated alkaline phosphatase and/or bilirubin levels can point the secondary etiology to primary sclerosing cholangitis (PSC), a condition with a reported association to pouchitis [14]. Stool studies should include *C. difficile* toxin, especially in cases of increased stool frequency. When coupled with systemic symptoms such as fever and chills, additional testing should include stool cultures (for *Salmonella, Shigella, Yersinia,* and *Campylobacter*) and *E. coli* O157:H7 specific testing [6]. Although not routinely obtained, celiac serology can be collected during initial evaluation [2]. In case of low total IgA or identification of anti-TTG IgA antibodies, IgG TTG and IgG deamidated gliadin peptides (DGPs) should be obtained. If their results are suspicious of celiac disease, an intestinal biopsy is needed for confirmation.

Endoscopic evaluation of the afferent limb and the proximal and distal pouch is the most important step for proper diagnosis, and pouch biopsy should always be performed (gross findings alone are nonspecific). This is especially true in cases of CARP, as an offending agent should be ruled out. Endoscopic findings can include diffuse erythema with or without edema, granularity, friability, bleeding, mucous exudates, erosions, and ulcerations [6]. If endoscopic findings are indicative of Crohn's, they should be correlated with histology and further imaging (i.e., bowel follow-through X-ray or computed tomography) [13].

Histology can present acute or chronic inflammation with infiltration of polymorphonuclear leukocytes, crypt abscesses, and ulceration. Although this has limited specificity and pouchitis grading ability, it can provide useful pathogenic features such as granulomas, dysplasia, inclusion bodies (indicating CMV), or ischemia [6]. In our case, histopathological studies yielded lymphocytic infiltration with villous atrophy, indicative of celiac disease.

To the best of our knowledge, very few cases of CARP have been associated with celiac disease, with only one case similarly describing de novo celiac development post-IPAA surgery [4, 15].

The diversity in clinical presentation, disease course, and histopathology of pouchitis makes it a diagnostic challenge.

### 3.3. Treatment and Management

Antibiotics are the mainstay in the treatment of both acute and chronic pouchitis, and probiotics have a role in maintenance to prevent relapse. The first-line therapy is oral metronidazole 250 mg 3 times daily for 1 week or oral ciprofloxacin 500 mg twice daily for 1 week; ciprofloxacin has been shown to reduce the PDAI score and symptoms more than metronidazole [13]. For chronic antibiotic-dependent cases, maintenance therapy is indicated, with rifaximin at 200 mg OD being widely used to achieve remission for extended durations [16]. In a randomized controlled trial, a multistrain high potency probiotic preparation given for 9 months maintained remission in 85% percent of the group compared to 0% in the placebo group [17].

In CARP patients with no evidence of secondary causes (PSC, celiac disease, Crohn's, etc.), antibiotic combination therapy might be necessary. Rifaximin at 1 g twice daily with ciprofloxacin 500 g twice daily each for 15 days was tested by Gionchetti et al. and proved effective [11]. In case of failure of combined antibiotic treatment, immunogenic CARP can be suspected. Therapy is then shifted to rectal mesalamine 1 g OD for 4 weeks (initial therapy), and then hydrocortisone suppository OD for 2 weeks [18].

Pouchitis patients found to have celiac disease must adhere to a life-long gluten-free diet, barring wheat, barley, and rye from their food products [19]. Regular monitoring for micronutrient deficiencies is recommended as a follow-up with their dietician, especially iron, folic acid, vitamin B12, and vitamin D [19].

## 4. Conclusion

This case illustrates the diagnostic challenges in managing CARP and the importance of considering secondary etiologies such as celiac disease, even in patients with no prior history of gluten sensitivity. The identification of de novo celiac disease in this patient following IPAA surgery highlights the need for a thorough and systematic approach to the initial evaluation of persistent symptoms in pouchitis. The successful resolution of symptoms with a gluten-free diet emphasizes the critical role of dietary management in cases where celiac disease is identified as a contributing factor. Ongoing monitoring and patient education remain vital to ensure long-term remission and prevent recurrence. This report adds to the growing body of evidence that secondary causes should be carefully considered in the management of CARP, paving the way for more personalized treatment strategies in this challenging patient population.

## Data Availability

Data will be made available upon request from authors.
